# Small Samples, Big Insights: A Methodological Comparison of Estimation Techniques for Latent Divergent Thinking Models

**DOI:** 10.3390/jintelligence13110150

**Published:** 2025-11-17

**Authors:** Selina Weiss, Lara S. Elmdust, Benjamin Goecke

**Affiliations:** 1 Institute of Psychology, University of Hildesheim, Universitätsplatz 1, 31141 Hildesheim, Germany; lara.elmdust@googlemail.com; 2 Hector Research Institute of Education Sciences and Psychology, University of Tübingen, 72074 Tübingen, Germany; benjamin.goecke@uni-tuebingen.de

**Keywords:** small sample, Bayesian SEM, creativity, divergent thinking

## Abstract

In psychology, small sample sizes are a frequent challenge—particularly when studying specific expert populations or using complex and cost-intensive methods like human scoring of creative answers—as they reduce statistical power, bias results, and limit generalizability. They also hinder the use of frequentist confirmatory factor analysis (CFA), which depends on larger samples for reliable estimation. Problems such as non-convergence, inadmissible parameters, and poor model fit are more likely. In contrast, Bayesian methods offer a robust alternative, being less sensitive to sample size and allowing the integration of prior knowledge through parameter priors. In the present study, we introduce small-sample-size structural equation modeling to creativity research by investigating the relationship between creative fluency and nested creative cleverness with right-wing authoritarianism, starting with a sample size of *N* = 198. We compare the stability of results in frequentist and Bayesian SEM while gradually reducing the sample by *n* = 25. We find that common frequentist fit indexes degrade below *N* = 100, while Bayesian multivariate Rhat values indicate stable convergence down to *N* = 50. Standard errors for fluency loadings inflate 40–50% faster in frequentist SEM compared to Bayesian estimation, and regression coefficients linking RWA to cleverness remain significant across all reductions. Based on these findings, we discuss (1) the critical role of Bayesian priors in stabilizing small-sample SEM, (2) the robustness of the RWA-cleverness relationship despite sample constraints, and (3) practical guidelines for minimum sample sizes in bifactor modeling.

## 1. Introduction

It is consensual to assume that creativity is a fundamental human competency, critical for academic achievement (Gajda et al. 2017), mental health outcomes (Taylor 2017), and possibly innovation across diverse real-world contexts. Recognized as a vital human resource in professional settings such as innovation-driven industries (e.g., technology, design, and R&D), creativity has consequently gained growing attention in educational research, with scholars increasingly investigating its role in school environments (Barbot et al. 2019; Barbot and Kaufman 2025). Within psychological research, divergent thinking (DT)—the ability to generate multiple, novel solutions to open-ended problems (Guilford 1967)—is widely accepted as a core indicator of creative potential (Runco and Acar 2012). Conceptualized as both a core component of the creative process and a robust predictor of creative potential (Lubart et al. 2010), DT also serves as a key marker of everyday creativity (Kaufman and Beghetto 2009). However, creativity research often encounters a methodological challenge: small samples, especially in specialized studies using cutting-edge technologies (e.g., virtual-reality paradigms (Barbot et al. 2023) or investigations of high-expertise populations using experience-sampling designs (Benedek et al. 2017). These constraints are amplified by complex test batteries—often involving open-ended tasks that require painstaking, expert-based scoring—which limit recruitment throughput.

Despite these constraints, creativity-related variables like DT are best understood as a latent ability (e.g., Weiss et al. 2021, 2024a), ideally assessed by leveraging modeling techniques like confirmatory factor analysis (CFA) or structural equation modeling (SEM). However, these frequentist methods are notoriously sensitive to sample size-raising issues such as non-convergence, inflated standard errors, and model instability in small sample size studies (van De Schoot and Miočević 2020). Indeed, although recent methodological advances (e.g., Huang et al. 2017) have yielded a range of analytic frameworks that can accommodate small sample sizes while still ensuring adequate statistical power, unbiased parameter estimates, and robust model fit, the literature still lacks a coherent definition of what constitutes a small sample size (Wolf et al. 2013).

As a result, such methods often underperform or fail outright under certain data constraints. Bayesian SEM, on the other hand, offers a promising alternative: by integrating prior information and relaxing asymptotic assumption, this method has demonstrated greater robustness with limited data availability (Depaoli 2021). However, it remains unclear at what point traditional models begin to “break down” and how Bayesian approaches compare across specific thresholds.

In the current study, we therefore address this research gap in the context of DT by introducing small sample size modeling to creativity research. To this end, we systematically compare frequentist and Bayesian estimation approaches using an application example: modeling the relationship between DT fluency and its subfacet of cleverness with right-wing authoritarianism (RWA), a trait arguably linked to cognition and creativity suppression (Iannello et al. 2025).

### 1.1. Why Is the Measurement of Divergent Thinking More Complex than Other Ability Measurements?

In conventional ability testing, each item is keyed to a single objectively correct (veridical) response that examinees must identify (Cronbach 1949). Because this response is predetermined, scoring proceeds dichotomously—assigning correct answers and incorrect ones—which then forms the basis for subsequent psychometric analyses. In DT assessments, the response space is inherently open-ended rather than fixed, reflecting the construct’s fundamentally unconstrained nature. As already stated in Guilford’s presidential address in front of the APA (Guilford, 1950), this absence of a veridical response is fundamental for the construct but leads to various challenges when it comes to test administration and especially scoring.

The Alternate Uses Task (AUT; Guilford 1950; Wallach and Kogan 1965)) is the most commonly used measure of DT in creativity research (Saretzki et al. 2024). Usually, the participants are either instructed to be fluent—emphasizing idea quantity (Runco and Acar 2010)—or to be creative, which stresses novelty and originality. In some cases, such as this study, a hybrid version of the instructions was used (“be-fluent-original”) (Forthmann et al. 2017). Fluency and originality thus represent two core dimensions of DT (Carroll 1993; Weiss et al. 2024b). Accordingly, DT is often scored along multiple dimensions, including fluency and originality, each with their own psychometric considerations (e.g., Weiss et al. 2024b). Competing scoring approaches add complexity to both measurement and analysis. In particular, different scoring components may reflect overlapping but distinct psychological processes, which complicates attempts to draw clear conclusions from DT data—especially in studies with limited sample sizes, where enough power might be desired.

### 1.2. Fluency and Cleverness

Building on the multidimensional nature of DT, it becomes clear that fluency alone offers an incomplete representation of creative ideation. Fluency, defined as the quantity of ideas generated in response to a problem (French et al. 1963), reflects broad retrieval ability within Carroll’s (1993) three-stratum theory of cognition (e.g., Goecke et al. 2025). Fluency’s ease of measurement has led many DT tasks to focus exclusively on this dimension (Benedek et al. 2014). However, reducing DT to mere fluency proves conceptually inadequate, as it disregards idea quality entirely (e.g., Forthmann et al. 2025).

However, capturing the qualitative part of an idea adds complexity. For example, originality—one key indicator of the qualitative aspect of a response in a DT task—itself comprises multiple subcomponents: uncommonness (statistical rarity), remoteness (associational distance from typical responses), and cleverness (imaginative or ingenious quality; Wilson et al. 1953). These dimensions align with the consensus definition of creativity as novel and meaningful (Runco and Jaeger 2012), though they primarily reflect novelty rather than usefulness. While all of these reflect novelty, only cleverness engages deeper cognitive processes such as insight, humor, and ingenuity—attributes often central to everyday conceptions of creativity (French et al. 1963). Notably, Stratton and Brown (1972) explicitly equated cleverness with creativity, while Sawyer et al. (2003) characterized cleverness as everyday creativity, which is frequently assessed through DT tasks (Kaufman and Beghetto 2009). Cleverness, therefore, captures a more nuanced, affectively resonant form of originality that may be undervalued in scoring approaches that rely purely on fluency or statistical rarity. Yet, because clever ideas still require a broad associative search, cleverness likely overlaps with general ideational fluency. This makes it an ideal candidate for latent modeling approaches that can disentangle shared and unique variance—such as bifactor models. For example, such complexity can be addressed by employing a bifactor (S-1) modeling framework (Eid et al. 2017a), specifying a general fluency factor to capture shared variance and a nested cleverness factor to isolate the unique contribution of cleverness. This approach simultaneously accounts for aggregation challenges in DT assessment (Barbot et al. 2019) and tests whether cleverness retains substantive meaning beyond its fluency-based components (Reiter-Palmon et al. 2019).

### 1.3. How Can We Handle Small Sample Sizes?

Investigating complex psychological relationships in small samples requires modeling techniques that can capture nuanced structures like in bifactor models without being derailed by small sample sizes. Traditional frequentist methods, such as maximum likelihood (ML) estimation in SEM, often struggle under small sample conditions, leading to convergence failures, unstable estimates, or underidentified models. Bayesian estimation methods offer a robust alternative. Many different reasons can convince a researcher to choose Bayesian estimation over the traditional frequentist approach. Depaoli (2021) describes the following main benefits: (1) handling complex models where frequentist estimators fail which is particularly relevant for small-N studies where traditional methods underfit or non-converge; (2) incorporating background information through priors, which stabilizes estimates when data are limited; (3) philosophical differences in statistical interpretation; and (4) direct applicability to small samples without relying on asymptotic assumptions. Crucially, by integrating prior information (e.g., derived from previous studies, expert knowledge, or meta-analytical evidence), Bayesian models can produce more stable estimates when data are lacking (Depaoli 2021).

Given our manuscript focuses on small-sample challenges, the following section first contrasts frequentist and Bayesian foundations, then details how Bayesian SEM specifically addresses low-N limitations through principled prior specification (see also Smid et al. 2020).

#### 1.3.1. Comparing Frequentist and Bayesian Estimation of Structural Equation Modeling

CFA is a statistical technique used to test whether measures of a construct align with the researcher’s understanding of its nature. It examines how well observed variables (items) represent hypothesized latent factors (Brown 2015). SEM extends CFA by allowing researchers to test complex networks of relationships among latent variables while accounting for measurement error. Together, these approaches provide a framework for testing theoretical models against empirical data (Eid et al. 2017a).

As is commonly known, frequentist estimation treats model parameters as fixed but unknown, using estimators like ML to derive point estimates. ML estimation aims to minimize a statistical criterion known as the fit function. This function quantifies the discrepancy between the observed sample covariances and the model-implied covariances for the same variables. The parameter estimates are derived by minimizing the squared differences between corresponding elements of these two covariance matrices (Kline 2023). In ML estimation, parameters are derived through iterative nonlinear optimization algorithms that progressively minimize the fit function. Evaluating model-data fit relies on comparing these predicted variances, covariances, or correlations with their observed counterparts, highlighting how each method captures explained variance differently (Mulaik 2009).

In frequentist statistics, uncertainty is expressed through confidence intervals, interpreted through asymptotic theory: a 95% CI indicates that 95% of similar intervals from repeated samples would contain the true parameter, without probabilistic claims about any specific interval (Brown 2015). Bayesian methods, however, treat parameters as random variables by incorporating prior distributions. This yields posterior distributions and credible intervals with direct probabilistic interpretations: a 95% CI [20, 60] implies a 95% probability the parameter lies within this range. Both approaches assume random data but diverge in parameter treatment. Frequentists rely on sampling distributions, while Bayesians update beliefs via posteriors (Depaoli and van de Schoot 2017).

The likelihood function serves as a common foundation for both frequentist and Bayesian SEM, yet its interpretation differs fundamentally between frameworks. In frequentist approaches, parameters are fixed quantities, and the likelihood estimates their values by treating data as random. Bayesian methods, conversely, treat parameters as random variables, combining the likelihood with priors to form posterior distributions (Greco et al. 2008). In SEM, where the true data-generating process is typically unknown, choices about distributions and functional forms inherently influence results. This underscores the need for transparency in justifying model assumptions (Agostinelli and Greco 2013).

The specification of prior distributions is a critical step in Bayesian SEM, requiring deliberate choices about distributional forms, hyperparameters, and informativeness levels. This iterative process combines background research, expert input, and prior predictive validation. In practice, different parameters demand distinct priors, e.g., normal distributions typically model means, factor loadings, and intercepts (Van De Schoot et al. 2018).

Prior strength exists on a continuum from diffuse—being minimally informative to weakly informative, which constrain plausible ranges to fully informative incorporating precise external knowledge. While informative priors can stabilize small-sample analyses, they may inadvertently dominate the likelihood if overly restrictive (Zondervan-Zwijnenburg et al. 2017). The Bayesian framework ultimately yields posterior distributions that fully characterize parameter uncertainty—a key advantage over frequentist point estimates (Gelfand and Smith 1990).

#### 1.3.2. Handling Small Samples with Bayesian SEM

Analyzing complex statistical models with small samples presents significant challenges in multivariate analyses, including SEM. Most SEM estimation methods rely on asymptotic assumptions and perform optimally with large samples. In smaller samples (*N* < 200), researchers often encounter convergence failures, inadmissible solutions (e.g., Heywood cases), or substantially biased parameter estimates (van De Schoot and Miočević 2020). This is particularly relevant in social and behavioral science, where data collection is frequently constrained by naturally small populations (Egberts et al. 2016) and difficult-to-access participant groups (Coleman et al. 2002). A review by (Russell 2002) demonstrated that approximately 40% of exploratory factor analyses used small samples with an *N* < 200. Similarly, methodological reviews found small samples (*N* < 100) in 18% of SEM applications (MacCallum and Austin 2000). Even in randomized trials, the average cluster size was just 29 in primary care research (Eldridge et al. 2004). While some researchers then resort to simpler techniques like multiple regression or manifest-variable path analysis to avoid issues related to complex statistical procedures like SEM, such approaches that rely on observed composite scores or single indicators rather than latent variables may actually exacerbate small-sample bias, particularly when failing to account for measurement error (van De Schoot and Miočević 2020).

These persistent challenges with small samples in traditional SEM approaches highlight the need for alternative estimation methods that are less reliant on large-sample assumptions. This is precisely where Bayesian estimation offers a promising solution: Unlike frequentist methods, it does not depend on asymptotic theory (Kaplan 2023), providing distinct advantages for small to moderate sample sizes. This robustness has been demonstrated across a range of statistical models, including mediation (e.g., Miočević et al. 2017), latent growth models (e.g., Zondervan-Zwijnenburg et al. 2019), multilevel structures (e.g., Baldwin and Fellingham 2013), autoregressive designs (e.g., Price 2012), and mixture models (e.g., Depaoli 2013). Of particular relevance to our study, simulation research specifically examining SEM supports these benefits. For instance, Lee and Song (2004) showed that Bayesian estimation produces stable results even with samples as small as *N* ≤ 75, whereas ML estimation often fails under these conditions—a finding corroborated by later work (Natesan 2015; van Erp et al. 2018). However, these studies consistently emphasize that Bayesian approaches outperform frequentist methods only when paired with well-specified prior distributions. Despite this evidence, applied researchers frequently default to software-generated priors, potentially undermining the method’s advantages (Van De Schoot et al. 2017). To ensure rigorous implementation, our study adheres to current methodological standards for prior specification, as detailed in the Methods section.

However, the existing evidence base is derived predominantly from simulation studies, with limited applications to empirical datasets (Smid et al. 2020). Accordingly, this study also aims to contribute to closing this research gap.

### 1.4. Application: The Case of Divergent Thinking and Right-Wing Authoritarianism

In the following, we describe a specific application case for small sample sizes in creativity research: The link between DT and Right-Wing Authoritarianism. The literature highlights a wide range of abilities and personality traits that have been linked to DT (e.g., Benedek et al. 2014; Weiss et al. 2021). Interestingly, despite the breadth of established nomological networks, ideological orientations, and particularly political ideologism, have received less attention as potential covariates of creativity and DT. While the broader concept of the *Political Person* has been extensively examined in relation to various psychological constructs (e.g., Ebert et al. 2023), its relevance for DT remains underexplored. This is surprising, since ideological beliefs can shape how persons deal with uncertainty, new ideas, and social norms; all of which are arguably related to creative thinking.

In studying ideological patterns and political personalities, RWA has been the focus of several studies. RWA reflects a cluster of traits including conventionalism, authoritarian submission, and aggression toward perceived deviants, which together predispose individuals toward rigid cognitive styles and resistance to unconventional ideas (Rubinstein 2003). RWA has also been related to low openness to experience (Butler 2000), which itself (openness) has been shown to have a stable relationship with DT (e.g., Benedek 2024; McCrae 1987). Additionally, a growing body of research suggests that RWA characteristics are meaningfully related to creativity, particularly DT: negative associations between RWA and various creativity-related measures are consistently reported, though the strength of these relationships varies across domains. For instance, work by Tyagi (2017) found a modest but significant negative correlation (*r* = −0.24) between RWA and self-reported creative personality traits, while more nuanced behavioral studies reveal even stronger effects. Silvia et al. (2021) demonstrated that individuals high in RWA generated humor productions rated as significantly less funny (*β* = −0.47), suggesting that authoritarianism may constrain the ability to engage in playful, unconventional ideation. This aligns with Dollinger (2007)’s findings that conservatism, which is closely related to RWA, correlated negatively (*r* = −0.22 to −0.27) with both real-world creative accomplishments and judged creativity of artistic products.

The mechanisms underlying these associations appear rooted in cognitive and motivational factors. Research by Iannello et al. (2025) highlights the role of cognitive flexibility, showing that individuals adept at shifting between perspectives—a hallmark of creative thinking—are less susceptible to dogmatic ideologies and better at evaluating complex information. Conversely, the rigid worldview associated with high RWA may inhibit the exploratory thinking necessary for creativity. An analysis of DT performance by Zmigrod et al. (2020) further clarifies this relationship: while partisan intensity (a proxy for ideological rigidity) was specifically linked to reduced flexibility on the AUT, other DT dimensions like fluency and originality showed weaker or nonsignificant associations. This implies that RWA’s constraining effects may target cognitive flexibility rather than impairing the sheer quantity of ideas. In practical terms, individuals high in RWA may struggle with generating diverse or unconventional ideas, even if they can produce many responses.

### 1.5. The Present Study

In this study, we aimed at introducing small sample size modeling to the field of creativity by comparing the performance of frequentist and Bayesian estimation methods under conditions of limited sample size. We evaluated how each approach handles model estimation as sample size decreases, focusing on robustness, convergence behavior, and parameter stability. This comparison was conducted within a CFA and a SEM framework, which are especially sensitive to small-N challenges.

To provide an application example for creativity research, we applied both estimation approaches to a bifactor model of DT that distinguishes between a general fluency factor and a nested cleverness factor. We then examined how RWA was associated with these aspects of DT. Based on previous findings, we expected stronger negative associations with cleverness than with fluency. This allowed us to assess not only the theoretical specificity of RWA–DT links, but also how robustly such effects can be estimated using different statistical frameworks across varying sample sizes. The study was not preregistered. We provide all data, materials, and scripts to reproduce the current results in the open science framework: https://osf.io/q5ap8.

In sum, our study investigates the following two-fold research aims using the application example of DT and its relationship to RWA:

(1) How are frequentist fit indices and Bayesian convergence diagnostics of a bifactor-(S-1)-model influenced by a gradual sample size reduction?
(2) How is RWA related to fluency and cleverness?

## 2. Methods

### 2.1. Sample and Resampling Procedure

This study conducted a secondary analysis of a dataset that was used in previous studies (Forthmann et al. 2017; Forthmann et al. 2020b; Forthmann and Doebler 2022)*.* The sample included *N* = 202 participants (women: *n* = 142; men: *n* = 60; age: *M* = 24.48, *SD* = 6.86), who were mainly university students (77.89%). To subsequently ensure the comparability of the estimation methods, as the handling of missing data was implemented differently in the procedures used, missing data was addressed through listwise deletion for all variables. This eliminated *n* = 2 cases. Furthermore, *n* = 2 outliers on the RWA variable, which were outside the interval of ±2 standard deviations around the mean, were removed due to their large influence on the model estimation (Eid et al. 2017b). This resulted in a final sample size of *N* = 198.

To systematically evaluate the robustness of both estimation methods under diminishing sample sizes, we employed a stepwise reduction procedure. Starting from the full sample (*N* = 198), we generated subsets by cumulatively removing 25 random observations without replacement at each step. For each reduced sample size, the target model was re-estimated 10 times using different random subsamples. This iterative resampling approach accounted for variability inherent in random data exclusion and provided more reliable estimates of performance trends. Across these iterations, we recorded the approximate fit indexes of interest, the multivariate Gelman-Rubin diagnostic (Rhat; Gelman and Rubin 1992), and the standard errors of the standardized parameters. The corresponding mean values were then extracted from the 10 iterations for each repetition.

### 2.2. Measures

DT was assessed with AUTs (Wallach and Kogan 1965) with three different objects (rope, garbage bag, paperclip). Participants were therefore instructed to be creative. Participants had 2.5 min to work on the tasks and were informed that there should be enough time to work on the tasks, which should make for a more relaxed situation (Forthmann et al. 2017). The tasks were scored for overall quality (subjective ratings based on uncommonness, remoteness, and cleverness), fluency (number of ideas), and complexity (aggregated score of the number of characters that were used for each of the ideas). In our study, we will capitalize on the scoring of fluency and cleverness. While fluency was displayed by a count of correct answers, the cleverness was rated on a five-point Likert scale by five human judges, with subsequent aggregation towards a mean per item across all five human judges (e.g., a mean cleverness score across all answers and judges for the item rope; see (Forthmann and Doebler 2022)). Cleverness has been understood as one indicator of originality (next to uncommonness and remoteness) that describes the aptness and thoroughness of responses (Forthmann and Doebler 2022; Wilson et al. 1953).

Additionally, some person-level covariates like verbal fluency, aspects of cultural diversity, and personality (e.g., aggression, submission, RWA) were measured. RWA was assessed using Zakrisson’s (2005) abbreviated scale. This 15-item measure comprises three subscales: aggression (4 items), conventionalism (6 items), and submission (5 items). The scale demonstrated marginally acceptable internal consistency in a sample of Forthmann and Doebler (2022), with Cronbach’s α = 0.67 (95% CI [0.60, 0.74]). Due to the scope of the manuscript, we only included RWA as a covariate in the present study.

### 2.3. Model Specification

**Figure 1:**
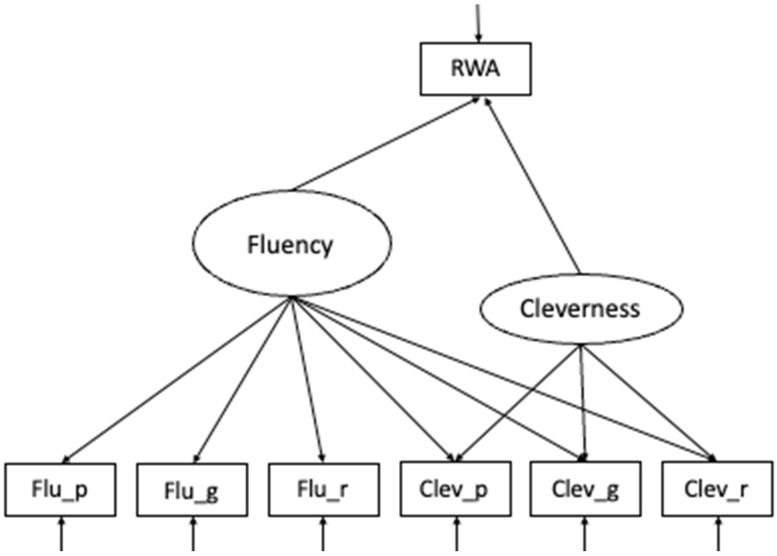
Schematic figure of the Bifactor (S-1) model of DT. Fluency indicators: *flu_p* = fluency paperclip, *flu_g* = fluency garbage bag, *flu_r* = fluency rope; Cleverness indicators: *clev_p* = cleverness paperclip, *clev_g* = cleverness garbage bag, *clev_r* = cleverness rope. RWA = Right-Wing Authoritarianism.

The target model included a measurement model for fluency and cleverness predicting observed RWA. All variables were included using z-standardization. As a targeted model, a Bifactor-(S–1) Model (Eid et al. 2017a, 2018) was estimated with the total sample size (Figure 1). The model comprised a specific factor for cleverness indicated by three items, and a general factor, capturing variance in creative fluency. In Bifactor modeling, the general factor (in this case, fluency) represents variance that is shared by all indicators, whereas the nested factor captures specific variance unique to a particular domain, ability, or scoring. In the present model (see Figure 1), the specific factor represented a narrower ability (cleverness), capturing variance unique to cleverness above and beyond variance explained by the general fluency factor.

To evaluate whether our sample size (*N* = 198) was adequate for the planned SEM, we conducted a Monte Carlo power analysis using the lavaan package in R. The population model, as displayed in Figure 1, included two latent factors, each defined by their respective indicators, with both factors predicting right-wing authoritarianism (RWA) via moderate standardized paths (β = 0.30). Across 1000 simulated samples, model convergence was 100%, and parameter recovery was excellent (bias < 0.006). The estimated power to detect the specified paths was 0.957 for RWA~fluency and 0.928 for RWA~cleverness, both exceeding the conventional 0.80 threshold. These results indicate that the available sample provided sufficient statistical power to detect effects of moderate size and to reliably estimate the proposed SEM model structure.

The model was estimated using two statistical frameworks—frequentist and Bayesian SEM —to compare their performance under varying sample sizes.

The frequentist approach was implemented using the *lavaan* package (Rosseel 2012) in R version 4.5.0. Parameters were estimated via maximum likelihood with robust standard errors (MLR). Missing values were handled using full information maximum likelihood (FIML), which leverages all available data points to produce unbiased estimates (Brown 2015).

The Bayesian equivalent was estimated using the *blavaan* package (Merkle and Rosseel 2018) in R version 4.5.0, which interfaces with Stan for Markov Chain Monte Carlo (MCMC) sampling. The implemented algorithm is the No-U-Turn Sampler (NUTS) as a form of the Hamiltonian Monte Carlo algorithm (HMC) (Merkle et al. 2021). The specification of prior distributions is a critical step in Bayesian analysis. For this study, we selected weakly informative priors. This approach provides mild regularization, constraining parameters to plausible ranges to stabilize estimation with limited data, without strongly influencing the results away from what the data suggest (Depaoli 2021; van de Schoot et al. 2017). The priors were chosen based on established conventions for standardized parameters in psychological measurement (e.g., Bürkner 2017; Merkle and Rosseel 2018).

Specifically, the priors listed in Table 1 were specified as follows:

Item and Factor Intercepts (nu, alpha): We used a normal(0, 10) prior. Given that our indicators were z-standardized, this prior is extremely wide and effectively non-informative, centering the intercepts at zero but allowing them to be freely estimated by the data. Factor Loadings (lambda): A normal(0, 2) prior was chosen. For standardized solutions, this is a conservative default that readily encompasses the range of typical factor loadings (approximately ±1) while applying minimal regularization to prevent the estimation of extreme and psychometrically implausible values (e.g., > |3|) in small samples. Regression Coefficients (beta): We specified a normal(0, 5) prior. This prior is wider than that for the factor loadings, reflecting our greater uncertainty about the magnitude of the structural relationships between RWA and the latent factors compared to the well-established measurement model. Covariances (rho): For the correlations between factors, we used a beta(2, 2) prior. This distribution is defined on the interval [0, 1] and is a natural choice for correlation matrices in Bayesian estimation, gently favoring values near zero but allowing for a wide range of positive correlations. Residual Variances (theta, psi): We employed an inverse-gamma(0.5, 0.5) prior (equivalent to gamma(0.5, 0.5) on the precision). This is a common, minimally informative prior for variance parameters that helps prevent the occurrence of Heywood cases (negative variances) in small-sample estimation.

This combination of priors represents a principled and widely accepted approach for stabilizing complex model estimation without requiring strong prior knowledge from previous studies.

The MCMC algorithm constructs the posterior distribution through iterative random sampling, similar to assembling a puzzle without a reference picture. Each drawn sample contributes to reconstructing the posterior distribution. This process offers some key advantages like flexible handling of complex models, explicit incorporation of prior knowledge through prior distributions, and comprehensive representation of parameter estimates as probability distributions (Depaoli 2021).

However, practical application of MCMC presents specific challenges. Convergence of Markov chains requires careful diagnosis using tools like trace plots. Gelman et al. (2013) caution against premature conclusions from short chains. A burn-in phase, where initial iterations are discarded, is essential to reduce dependence on starting values. Therefore, this study employed 3 chains, each with 1000 iterations after a burn-in period of 500, which corresponds to the default in *blavaan* (Merkle and Rosseel 2018). Convergence was assessed using multivariate Rhat. Posterior summaries, including means, medians, and 95% highest posterior density (HPD) intervals, were computed for all parameters.

### 2.4. Fit Evaluation

The study evaluated model performance through three primary lenses: (1) frequentist fit indices, (2) Bayesian convergence diagnostics, and (3) comparative precision of parameter estimates across sample sizes. For frequentist analysis, we examined changes in established approximate fit indices—the Comparative Fit Index (CFI), Root Mean Square Error of Approximation (RMSEA), and Standardized Root Mean Square Residual (SRMR)—as sample size decreased. The CFI, an incremental fit index ranging from 0 to 1, quantifies the proportional reduction in misfit relative to a baseline model, with values approaching 1 indicating excellent fit (Brown 2015). Unlike parsimony-adjusted indices, the CFI does not penalize model complexity (Bentler 1990). In contrast, the RMSEA represents an absolute badness-of-fit measure that scales misfit by degrees of freedom, with lower values (ideally < 0.06) indicating better fit. Its reliance on noncentral chi-square distributions makes it sensitive to misspecification in complex models (Steiger 1990). The SRMR, another absolute fit index, calculates the average discrepancy between observed and model-implied correlations, where zero denotes perfect fit (Jöreskog and Sörbom 1981). Unlike the CFI or RMSEA, it depends solely on residual correlations rather than sample size or model complexity (Pavlov et al. 2020).

While traditional cutoff criteria (e.g., CFI > 0.90, RMSEA < 0.08) have been widely cited (Bentler and Bonett 1980; Browne and Cudeck 1989), their universal applicability has been challenged. Hu and Bentler (1999) proposed stricter thresholds (CFI > 0.95, RMSEA < 0.06, SRMR < 0.08) to minimize Type I/II errors, though subsequent work cautions against rigid cutoffs due to their sensitivity to sample size and model characteristics (Marsh et al. 2004). For our study, these indices served not as binary fit judgments but as tools to track relative changes across sample reductions.

For Bayesian estimation, we assessed convergence via Rhat, which compares within-chain and between-chain variance across multiple Markov chains (Gelman and Rubin 1992). Values of Rhat ≤ 1.05 indicate stable convergence, implying chains have forgotten their initial values and sufficiently explored the posterior distribution (Vehtari et al. 2021). This approach mirrors ANOVA logic, partitioning total variance into within-chain and between-chain components to quantify mixing efficiency. Reliable inference requires that chains converge to a common stationary distribution, evidenced by negligible differences in their variance estimates (Gelman et al. 2013).

## 3. Results

### 3.1. Right-Wing Authoritarianism, Fluency, and Cleverness in the Full Sample

The confirmatory factor analysis of our measurement model demonstrated acceptable fit to the data: χ^2^(21) = 23.20, *p* = .001, CFI = 0.96, RMSEA = 0.12, and SRMR = 0.04. Standardized factor loadings revealed significant indicators for both latent factors, except “cleverness rope” for fluency (*r* = 0.147, *SE* = 0.080, *z* = 1.829, *p* = 0.067, 95%-CI = [−0.010, 0.304]).

First, we investigated how RWA is related to fluency and cleverness in the full sample. Based on the frequentist estimation, we found the SEM as displayed in Figure 1 to fit the data acceptably well: χ^2^(25) = 33.66, *p* < .001, χ^2^/df = 1.35, CFI = 0.93, RMSEA = 0.11, and SRMR = 0.04. In Table 2, we reported the factor loadings, the standard errors, the z-value, the *p*-value, and the 95% confidence interval of the manifest items.

All indicators except for “cleverness rope” were explained by the general fluency factor. It can be observed that the fluency items load higher on the general fluency factor than the cleverness items, which, in contrast, exhibit a substantially stronger loading of these items on the nested creative cleverness factor.

At the structural level, only the relationship between creative cleverness and RWA was significant (*r* = −0.359, *SE* = 0.075, *z* = −4.821, *p* < .001, 95%-CI = [0.506, 0.213]), while fluency showed no significant association with RWA (*r* = −0.013, *SE* = 0.072, *z* = −0.175, *p* = .861, 95%-CI = [−0.154, 0.129]).

The Bayesian estimation of our measurement model showed excellent convergence (Multivariate Rhat = 1.000) with all parameter-specific Rhat-values ≤ 1.001, indicating stable posterior distributions. All credible intervals of the factor loadings excluded zero except for the cross-loading of clev_r on the general factor (λ = 0.15, 95%-CI = [0.00, 0.30]). In comparison to the frequentist model, we investigated the full model based on Bayesian estimations. The Rhat-values indicated convergence of chains (Multivariate Rhat = 1.001). In Table 3, we reported the Rhat, the median and mean of the loadings, the standard errors, and the 95%-credible interval of the manifest items.

All manifest items had credibility intervals that did not include zero. Replicating the MLR results, fluency items loaded higher on the general factor (Range_Mean_ = [0.699, 0.865]) than the cleverness items (Range_Mean_ = [0.149, 0.339]) while these items loaded higher on the nested factor (Range_Mean_ = [0.614, 0.676]). However, Bayesian estimates showed greater precision, with narrower posterior distributions for both the general (Range_SE_ = [0.035, 0.076]) and the nested factor (Range_SE_ = [0.062, 0.063]) relative to MLR standard errors.

At the structural level, the CI for the relationship between fluency and RWA included zero (Rhat = 1.000, median = −0.012, mean = −0.014, SE = 0.079, 95%-CI = [−0.168, 1.141], HDI = [−0.167, 0.132). In contrast, the creative cleverness RWA link matched the MLR estimate in both magnitude and precision (Rhat = 1.000, median = −0.348, mean = −0.357, SE = 0.078, 95%-CI = [−0.509, −0.205]).

### 3.2. How Is the Model Fit Influenced by a Gradual Sample Size Reduction?

**Figure 2:**
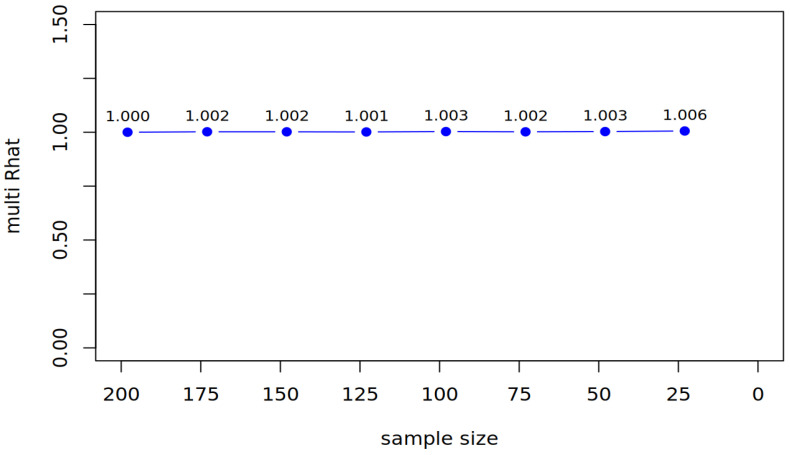
BayesT: The change in the multivariate Rhat.

To evaluate how progressively smaller samples affect model fit and parameter precision, we reduced the sample size in steps of 25 participants (from *N* = 198 to *N* = 23). At each step, we compared frequentist (MLR) and Bayesian (BayesT) estimations in terms of fit indices, factor loadings, and standard errors. Table 4 and Figure 2 display the fit indices for all reduction steps for both methodological approaches. Additional fit indices (Frequentist: RMSEA, SRMR) and detailed parameter uncertainty trends are provided in Appendix A.

#### 3.2.1. Reduction by 25 Participants

After reducing the sample to *N* = 173, the frequentist estimation yielded an acceptable fit, with similar loading patterns, like in the full sample (for an overview of fit and loading patterns, see Table 4). The Bayesian estimation showed good convergence (see Figure 2: Multivariate Rhat = 1.001). Again, fluency items loaded higher on the general factor (RangeMean = [0.695, 0.863]) compared to cleverness items (RangeMean = [0.145, 0.334]), while cleverness items exhibited stronger loadings on the nested factor (RangeMean = [0.608, 0.672]). The Bayesian estimates demonstrated greater precision, with narrower posterior distributions for both factors (general: RangeSE = [0.036, 0.077]; nested: RangeSE = [0.061, 0.064]). In Table 5, we display the standard errors and how they changed from the full sample to the first reduction (Δ_1_) for all manifest items in both estimation methods.

At the structural level in the frequentist approach, the relationship between creative cleverness and RWA remained significant (*r* = −0.351, *SE* = 0.078, *z* = −4.50, *p* < .001, 95%-CI = [−0.504, −0.198]), whereas fluency showed no significant association with RWA (*r* = −0.015, *SE* = 0.075, *z* = −0.20, *p* = .841, 95%-CI = [−0.162, 0.132]). Compared to that in the Bayesian approach, the credible interval for the fluency–RWA relationship included zero (Rhat = 1.000, median = −0.013, mean = −0.016, SE = 0.082, 95%-CI = [−0.176, 0.148]). The creative cleverness–RWA link remained significant (Rhat = 1.000, median = −0.342, mean = −0.350, SE = 0.081, 95%-CI = [−0.508, −0.192]).

#### 3.2.2. Reduction by 50 Participants

After reducing the sample to *N* = 148, the frequentist estimation yielded an acceptable fit and loading patterns like in the first reduction step (for an overview of fit and loading patterns, see Table 4). The Bayesian estimation demonstrated good convergence (see Figure 2: Multivariate Rhat = 1.002), with loading patterns similar to the first reduction step. Bayesian estimates again showed greater precision, with narrower posterior distributions (general factor: RangeSE = [0.038, 0.079]; nested factor: RangeSE = [0.063, 0.066]). In Table 5, we display the standard errors and how they changed from the first to the second reduction and the cumulative change in the standard error (Δcum) for all manifest items in both estimation methods. For the second reduction step, both estimators exhibited comparable increases in standard errors.

At the structural level in the frequentist approach, the negative association between creative cleverness and RWA remained significant (*r* = −0.345, *SE* = 0.082, *z* = −4.21, *p* < .001, 95%-CI = [−0.505, −0.185]). Fluency again showed no significant link to RWA (*r* = −0.018, *SE* = 0.079, *z* = −0.23, *p* = .819, 95%-CI = [−0.173, 0.137]). Compared to that in the Bayesian approach, the credible interval for fluency and RWA included zero (Rhat = 1.000, median = −0.017, mean = −0.019, SE = 0.085, 95%-CI = [−0.185, 0.151]). The creative cleverness–RWA relationship remained significant (Rhat = 1.000, median = −0.338, mean = −0.346, *SE* = 0.084, 95%-CI = [−0.510, −0.181]).

#### 3.2.3. Reduction by 75 Participants

After reducing the sample to *N* = 123, the frequentist estimation yielded an acceptable fit and loading patterns like in the other reduction steps (for an overview of fit and loading patterns see Table 4). Similarly, the Bayesian estimation demonstrated excellent convergence (see Figure 2: Multivariate Rhat = 1.001). Fluency items loaded robustly on the general factor (RangeMean = [0.680, 0.854]), while cleverness items had lower general factor loadings (RangeMean = [0.130, 0.324]) but higher nested factor loadings (RangeMean = [0.595, 0.660]). Bayesian estimates showed greater precision for fluency items. Again, in Table 5, we display the standard errors and how they changed from the second to the third reduction, as well as the cumulative change in the standard error (Δcum) for all manifest items in both estimation methods. For fluency items, the increase in standard errors was smaller with the Bayesian method—for instance, “fluency paperclip” showed a minimal change (ΔBayesT = 0.004) compared to the MLR estimator (ΔMLR = 0.010). However, results were mixed for cleverness items, as seen with the cleverness paperclip, where the Bayesian SE (0.093) was slightly lower than the MLR SE (0.106).

At the structural level in the frequentist approach again, the negative association between creative cleverness and RWA remained significant (*r* = −0.340, *SE* = 0.085, *z* = −4.00, *p* < .001, 95%-CI = [−0.506, −0.174]). Fluency again showed no significant link to RWA (*r* = −0.020, *SE* = 0.082, *z* = −0.24, *p* = .807, 95%-CI = [−0.181, 0.141]).

In the Bayesian approach, the credible interval for fluency and RWA included zero (Rhat = 1.000, median = −0.019, mean = −0.022, *SE* = 0.088, 95%-CI = [−0.194, 0.154]). The creative cleverness–RWA relationship remained significant (Rhat = 1.000, median = −0.335, mean = −0.342, *SE* = 0.087, 95%-CI = [−0.512, −0.173]).

#### 3.2.4. Reduction by 100 Participants

After reducing the sample to *N* = 98, the frequentist estimation yielded an acceptable fit and loading patterns like in the other reduction steps (for an overview of fit and loading patterns, see Table 4). Similarly, the Bayesian estimation demonstrated excellent convergence (see Figure 2: Multivariate Rhat = 1.001) and similar loading patterns compared to the previous reduction. Bayesian estimates demonstrated slightly better precision for most items (e.g., flu_p: SE = 0.045 vs. MLR SE = 0.054). Again, in Table 5, we display the standard errors and how they changed from the third to the fourth reduction, as well as the cumulative change in the standard error (Δcum) for all manifest items in both estimation methods. In the fourth reduction step, both estimators demonstrated similar patterns of standard error increases, though with some notable differences: For fluency items, Bayesian standard errors remained consistently smaller.

At the structural level, both approaches performed comparably to the previous reduction steps (Cleverness and RWA frequentist: *r* = −0.335, *SE* = 0.090, *z* = −3.72, *p* < .001, 95%-CI = [−0.511, −0.159]; Cleverness and RWA Bayesian: Rhat = 1.000, median = −0.330, mean = −0.338, *SE* = 0.091, 95%-CI = [−0.516, −0.161]).

#### 3.2.5. Reduction by 125 Participants

After reducing the sample to *N* = 73, we encountered convergence issues, with negative variances emerging for observed variables. To address this, we implemented parameter bounds in the CFA command to constrain variance estimates. Using these bounds, the frequentist model yielded an acceptable fit and loading patterns like in the other reduction steps (for an overview of fit and loading patterns, see Table 4). The Bayesian estimation demonstrated excellent convergence stability, as indicated by a near-perfect multivariate Rhat value of 1.002 (see Figure 2). Notably, the analysis required no parameter constraints, highlighting the method’s robustness in small-sample conditions. Again, in Table 5, we display the standard errors, the change in the standard error from the fourth to fifth reduction, and the cumulative change in the standard error for the manifest items for both estimators. In terms of precision, the Bayesian approach demonstrated clear advantages for fluency items, producing standard errors 40–50% smaller than MLR (e.g., “fluency paperclip”: MLR *SE* = 0.097 vs. BayesT *SE* = 0.051). However, both methods showed comparable increases in standard errors for cleverness items (Δ*SE* ≈ 0.015–0.020).

The structural relationships showed that creative cleverness retained a significant negative association with RWA (*r* = −0.325, *SE* = 0.095, *z* = −3.42, *p* < .001, 95%-CI = [−0.511, −0.139]) and that fluency remained nonsignificant (*r* = −0.025, *SE* = 0.093, *z* = −0.27, *p* = .787, 95%-CI = [−0.207, 0.157]). The structural relationships closely mirrored frequentist results, but with the advantage of more precise interval estimates. Specifically, the creative cleverness-RWA path showed a median coefficient of −0.320 (95%CI = [−0.515, −0.125]), while the non-significant fluency-RWA association was weaker (median = −0.023, 95%-CI = [−0.210, 0.164]).

#### 3.2.6. Reduction by 150 Participants

The reduction to *N* = 48 represented a critical test of both estimation methods’ robustness under extreme sample size constraints. The frequentist MLR estimation, despite requiring parameter bounds to avoid negative variances, produced marginally acceptable fit indices (see Table 4). In contrast, the Bayesian estimation exhibited remarkable stability, achieving convergence (see Figure 2: Multivariate Rhat = 1.002) without requiring parameter constraints. The method demonstrated particular advantages for estimating general fluency parameters, where standard errors were 35–45% smaller than their MLR counterparts (e.g., fluency paperclip SE = 0.061 vs. 0.093). This precision advantage was less pronounced for cleverness items, suggesting differential sensitivity to data sparsity across construct domains. In Table 5, we report the standard errors, the change in the standard error from the fifth to the sixth reduction, and the cumulative change in the standard error for the manifest items for both estimators. Notably, the comparison revealed divergent precision patterns across measurement domains. For fluency indicators, BayesT’s cumulative standard error increases (Δ = [0.026, 0.039]) were markedly smaller than MLR’s (Δ = [0.051, 0.064]), consistent with its theoretical advantages in small samples. However, this pattern reversed for cleverness items on the nested factor, where MLR unexpectedly showed smaller cumulative SE inflation (0.029–0.067) compared to BayesT (0.060–0.076). This counterintuitive result likely reflects the complex interplay between prior distributions and limited likelihood information at very small sample sizes.

At the structural level, the negative association between creative cleverness and RWA persisted, while creative fluency continued to show no meaningful relationship. The structural relationships in the Bayesian estimation mirrored frequentist results but with characteristically narrower credible intervals (creative cleverness-RWA: median = −0.305, 95%-CI = [−0.560, −0.050]; fluency-RWA: median = −0.025, 95%-CI = [−0.245, 0.195]).

#### 3.2.7. Reduction by 175 Participants

The final reduction to *N* = 23 pushed both estimation methods beyond their operational limits. The frequentist MLR estimation failed to produce admissible solutions despite implemented parameter bounds, with negative variance-covariance matrices occurring across all iterations. The Bayesian approach demonstrated greater resilience but ultimately reached its practical limits. Initial runs showed convergence failures (see Figure 2: Multivariate Rhat values up to 1.09) despite extended burn-in periods and chain lengths. After excluding two non-convergent samples, the remaining eight iterations suggested extreme parameter uncertainty: standard errors for cleverness items inflated dramatically (range = 0.167–0.269), while fluency items showed more moderate but substantial increases (range = 0.095–0.141). The structural relationships became essentially uninterpretable, with the creative cleverness-RWA association showing implausibly wide credible intervals (median = −0.295, 95% CI = [−0.750, 0.160]).

## 4. Discussion

In the present study, we aimed to introduce small-sample-size structural equation modeling to creativity research by applying it to the relationship between RWA and two aspects of DT—fluency and cleverness. This application example provides tentative methodological guidance for creativity researchers working with limited statistical power.

### 4.1. What Happens if Sample Sizes Are Small?

Our study makes three key methodological contributions to small-sample SEM in divergent thinking research: (1) benchmarking the performance of frequentist and Bayesian methods under systematic sample reduction, (2) illustrating how the chosen bifactor-(S-1)-model performs in terms of estimation stability across varying sample sizes, and (3) identifying practical thresholds for applied researchers.

Contrary to common assumptions about small-sample limitations, frequentist CFA performed adequately down to N = 100, with fit indices (CFI > 0.90, RMSEA < 0.10) meeting traditional thresholds (Kenny 2024). Only below *N* = 73 did convergence issues necessitate parameter constraints. However, standard errors inflated progressively (e.g., +40–50% for fluency items at *N* = 48), underscoring the trade-off between admissibility and precision in smaller samples. It is important to note that our model was relatively small, comprising only six indicators. This limited complexity likely contributed to the more stable estimation performance observed at smaller sample sizes and should be considered when generalizing these findings to larger, more complex models.

This resilience is partly attributable to our bifactor-(S-1)-structure, where the reference indicators (fluency items with single loadings on the general factor) anchor estimation. Bayesian SEM leveraged this structure more effectively: at N = 48, it reduced standard errors for reference indicators by 30–40% compared to MLR, while maintaining convergence without constraints. In contrast, the nested cleverness factor—which inherently carries greater parametric uncertainty due to its residualized nature (Eid et al. 2017a)—showed comparable instability across methods (ΔSE ≈ 10–15% increase). This aligns with simulation evidence that nested factors are more sensitive to sample size, regardless of estimator choice (Zondervan-Zwijnenburg et al. 2019).

The divergence in performance between fluency and cleverness stems not from construct clarity but from their distinct roles in the bifactor framework. Reference indicators (fluency) benefit from direct constraints on the general factor, whereas nested factors (cleverness) must disentangle shared and unique variance—a process inherently prone to propagation of error (Eid et al. 2018). For instance, in alternative model specifications (e.g., correlated factors), cleverness might show better reliability, but this would forfeit the bifactor’s theoretical advantage: isolating domain-general and domain-specific variance.

In summary, while Bayesian SEM offers robust advantages for small-sample bifactor models—particularly in stabilizing estimates through reference indicators—its effectiveness is inherently tied to the underlying theoretical design of the model. For instance, if the bifactor structure is mis-specified or if reference indicators are poorly chosen, even Bayesian estimation cannot fully compensate for these flaws. Thus, the method’s strengths are constrained by decisions about model architecture, factor definitions, and the alignment of indicators with theoretical constructs. Moving forward, researchers should pair methodological innovations (e.g., Bayesian small-sample techniques) with deliberate, theory-driven model specification. This means selecting models—whether bifactor, correlated factors, or another framework—based on their fit to the research question, rather than defaulting to statistical convenience.

### 4.2. What Researchers Should Consider Working with Small-Sample Sizes

The methodological insights from our study yield concrete recommendations for small-sample SEM, though researchers must consider model complexity as a critical moderator. Overall, we recommend that researchers carefully consider the trade-offs between measurement breadth and statistical power when designing small-sample divergent thinking studies.

As a foundational principle, the participant-to-parameter ratio should guide design choices more than absolute sample sizes alone (Kenny 2024). For reference, our bifactor-(S-1)-model with 6 indicators and 25 parameters showed stable estimation under the following conditions:

For models of comparable complexity (participant-to-parameter ratio ≥ 6:1), traditional MLR remains viable with 150+ participants, producing reliable fit indices (CFI > 0.90, RMSEA < 0.10) when normality corrections are applied. However, this threshold assumes moderate model complexity—larger models with cross-loadings or residual covariances may require higher ratios. Researchers should always verify convergence and check for Heywood cases, as these signal model misspecification rather than mere sample size limitations.

When the ratio falls between 2:1 and 6:1 (approximately 50–150 participants for our model), Bayesian SEM with carefully specified priors provides superior stability. The benefits are most pronounced when prior distributions incorporate empirical knowledge from meta-analyses or established measurement literature (e.g., fluency factor loadings in divergent thinking tasks). Weakly informative priors offer a balanced approach, constraining parameters to plausible ranges while letting data dominate estimation.

While our findings suggest that Bayesian methods face fundamental limitations below a 2:1 participant-to-parameter ratio (typically *N* < 50 for our model), the generalizability of this threshold warrants careful consideration. The robustness of small-sample Bayesian estimation depends on multiple factors, including model complexity (e.g., number of indicators per factor), prior specification (weakly vs. strongly informative), and effect sizes. Importantly, these limitations are not absolute but rather exist on a continuum—while Bayesian approaches generally outperform frequentist methods in small samples, their effectiveness diminishes as models become more complex relative to sample size. For researchers working with such constrained samples, we recommend two complementary strategies: First, model simplification through either reducing latent factors (e.g., using a unidimensional rather than bifactor approach) or creating composite scores for well-established constructs. Second, intensive longitudinal designs with ≥5 measurement occasions per construct (Zondervan-Zwijnenburg et al. 2019) can effectively increase the observations per parameter through repeated measures. These approaches are particularly valuable for studying hard-to-access populations (e.g., clinical groups, experts) or conducting pilot investigations where small samples are unavoidable. Ultimately, researchers should view these guidelines as flexible boundaries rather than rigid rules, adapting them to their specific theoretical and methodological context.

Regardless of the chosen analytical approach, we cannot overemphasize the importance of transparent and comprehensive reporting practices in small-sample research. Following Depaoli and van de Schoot’s (2017) WAMBS-Checklist, studies using Bayesian Estimators should always include: (1) detailed documentation of prior distributions and their justification; (2) full reporting of convergence diagnostics including Rhat values for all parameters; (3) thorough prior sensitivity analyses demonstrating how results vary under alternative prior specifications; and (4) complete reporting of standard errors or credible intervals across all estimated parameters. Even though we have to note that, in our study, we did not conduct a formal prior sensitivity analysis. We employed a single set of weakly informative priors, selected to ensure model identifiability and stable convergence across varying sample sizes. While the close correspondence between Bayesian and frequentist estimates suggests that these priors did not substantially bias the results, we acknowledge that systematically examining alternative prior specifications would provide a more comprehensive assessment of model robustness. Future studies employing Bayesian SEM, particularly in small-sample contexts, are encouraged to conduct formal sensitivity analyses to evaluate the influence of prior choices on parameter estimates and model fit.

### 4.3. Application: Are Individuals High on Right-Wing Authoritarianism Less Clever?

In order to provide an application example for small sample size modeling in creativity research, we used a theoretically based measurement model for fluency and cleverness and then related these factors to a manifest score of RWA. We opted to use a manifest score rather than modeling RWA as a latent construct, as the primary focus of the study was on the measurement and modeling of the DT components. Modeling RWA latently would have required additional indicators and increased model complexity, potentially obscuring the methodological comparison between estimation approaches that was central to our research aims. Given the theoretical and empirical closeness of fluency and cleverness, we chose a bifactor-(S-1)-model that included a general factor of fluency and a nested factor capturing shared residual variance in indicators of cleverness. The bifactor structure of our model draws strong theoretical support from Eid et al.’s (2017a) framework, which advocates for such representations to disentangle shared and unique variance in multidimensional constructs. Empirically, our results mirror Forthmann et al.’s (2017, 2020a) observation that fluency and originality are mechanistically intertwined: arguably, individuals who generate more responses (higher fluency) inherently have greater opportunity to produce clever solutions through combinatorial processes. This parallels classic findings in writing research by Burleson et al. (1987), where sheer verbal output (word count) mediated the relationship between writing speed and conceptual complexity, suggesting a fundamental cognitive principle whereby quantitative productivity enables qualitative breakthroughs (Forthmann et al. 2025).

The bifactor model revealed that the general fluency factor did not significantly predict RWA scores, whereas the nested cleverness factor—representing the unique variance in the cleverness indicators after accounting for the general fluency factor—showed a robust negative association with RWA. This indicates that only the variance in cleverness unrelated to general fluency was associated with lower RWA scores. Given the cross-sectional nature of our data, these results should not be interpreted causally; rather, they reflect patterns of statistical association. Our findings are consistent with prior work linking higher ideological rigidity to lower cognitive flexibility (Zmigrod et al. 2020) and reduced qualitative creativity (Silvia et al. 2021). Extending Dollinger’s (2007) research, we observed that higher cleverness scores predicted lower RWA, independent of general fluency, highlighting a selective link between qualitative aspects of creative ideation and RWA.

These findings carry two theoretical implications that advance our understanding of creativity and, furthermore, RWA. First, our results provide robust evidence for the structural validity of nested divergent thinking models. Most previous studies focused on fluency and originality, while cleverness was rarely assessed. The bifactor approach successfully disentangles cleverness as a distinct dimension beyond general fluency variance, empirically supporting its use as a methodological framework in future creativity research—particularly when investigating nuanced relationships. Second, while fluency showed no significant predictive effect on RWA, higher scores on the unique cleverness factor were associated with lower RWA scores. This pattern indicates that variation in the qualitative aspects of creative ideation was linked to variation in RWA, whereas the quantity of ideas (fluency) was not. Given the cross-sectional design, these findings reflect statistical associations rather than causal effects.

### 4.4. Limitations and Future Directions

The current study’s findings must be interpreted in light of several important limitations that simultaneously point toward valuable future research directions. Beginning with the measurement of cleverness, our modest reliability estimates (Forthmann and Doebler 2022) reflect broader challenges that have been well-documented in similar areas like assessing creative originality (Weiss et al. 2021). The subjective nature of cleverness ratings, while standard practice in creativity research, introduces unavoidable rater biases and contextual dependencies that may have influenced our results (Barbot et al. 2019). Furthermore, our exclusive focus on the AUT may have constrained our ability to capture the full spectrum of cleverness manifestations, as different divergent thinking tasks are known to elicit varying aspects of creative quality depending on the instructions (e.g., Weiss et al. 2024b). The relatively limited number of cleverness indicators in our measurement model, despite our use of a latent variable approach, should be further addressed in future studies.

Moving to the issue of construct interdependence, earlier research identified a robust correlation between fluency and cleverness raising fundamental questions about their discriminant validity that warrant careful consideration. This finding aligns with previous work by Jung et al. (2015) demonstrating that originality scores often reflect ideational quantity to a significant degree, as well as Forthmann et al.’s (2017) observations about shared cognitive mechanisms between fluency and originality. Several plausible explanations for this interdependence merit discussion. First, cleverness may emerge largely as a byproduct of fluency through simple probability. Generating more ideas naturally increases the chances of producing particularly clever responses (e.g., Forthmann et al. 2025). Second, both constructs may draw on common cognitive resources such as working memory capacity or cognitive flexibility. Third, and perhaps most importantly, current measurement approaches may inadequately separate these dimensions in ways that future methodological innovations could improve.

Regarding sample representativeness, our use of a student sample (although common in creativity research (Rubinstein 2003)) introduces several constraints on the generalizability of our findings. Most notably, our sample contained limited representation of individuals with high RWA tendencies, which may have attenuated the observed effects. The restricted age range also precluded developmental perspectives that could enrich our understanding of how these relationships manifest across the lifespan. Additionally, the cultural homogeneity of our sample leaves important questions about cross-cultural variability in creativity–authoritarianism relationships unanswered, but future research should explore such questions.

A further methodological limitation concerns the treatment of our observed variables. The RWA scale and the cleverness ratings are technically ordinal. While treating them as continuous is a common and often reasonable approximation in SEM, particularly with robust estimators like MLR and when the primary goal is a comparative methodological analysis, it remains a simplification. Future studies could build upon our work by employing estimators specifically designed for ordinal data, such as WLSMV in a frequentist framework or Bayesian models with ordered probit likelihoods, to confirm the robustness of the findings.

Furthermore, our decision not to include residual correlations between parallel indicators (e.g., between cleverness scores for different AUT objects) in the final bifactor-(S-1)-model can be challenged. We excluded the residual correlations due to fit issues. However, this specification choice warrants discussion for two reasons: First, some methodological work suggests that residual correlations between parallel indicators may reflect task-specific variance rather than mere measurement error. Second, their exclusion could potentially lead to inflated factor correlations in alternative model specifications. That said, our primary conclusions remain robust because the bifactor-(S-1) framework explicitly accounts for shared variance through its general factor. Future studies with larger samples should systematically compare these specifications to determine whether task-specific residual covariances meaningfully affect DT measurement.

Our comparative analysis of frequentist and Bayesian approaches with diminishing sample sizes suggests several promising avenues for advancing small-sample modeling techniques. First, future studies should systematically test how different model specifications (e.g., bifactor vs. higher-order structures) perform under small-sample conditions. Of particular interest would be examining whether more parsimonious structures (e.g., second-order models) might outperform bifactor models when samples are severely constrained. Second, the development of empirically grounded prior distributions for DT research could enhance Bayesian small-sample analyses. Meta-analytic studies of DT factor loadings across populations could inform more accurate weakly informative priors. Third, combining Bayesian estimation with regularization techniques (e.g., Bayesian LASSO) might help manage parameter inflation in complex models with small samples. Fourth, future work should establish minimum sample size guidelines for different model complexities in DT research, similar to Wolf et al.’s (2013) simulations for general SEM applications.

Lastly, our study operated within the SEM framework, which treats test scores or item parcels as indicators. A promising direction for future research lies in the application of Item Response Theory (IRT) models. IRT provides a richer measurement model by directly modeling responses at the item level, allowing for the estimation of parameters such as item difficulty (e.g., how “difficult” it is to generate a clever idea for a specific object) and discrimination. This is particularly relevant for divergent thinking tasks where the properties of different prompts can vary significantly. The integration of Bayesian estimation with IRT (e.g., Zhu et al. 2021) would be a possible extension of our work. These methodological advancements should be pursued alongside substantive investigations of the DT-RWA relationship. Future research directions emerging from our findings include developing multi-method assessment strategies that combine human ratings with scoring by large language models (e.g., Saretzki et al. 2025). For sample representativeness, targeted sampling of ideologically diverse populations, cross-cultural replications using standardized measures, and lifespan approaches examining developmental trajectories would all significantly advance this line of research. Ultimately, incorporating real-world DT measures alongside laboratory assessments may prove particularly valuable for enhancing ecological validity while maintaining methodological rigor.

Future studies could further illuminate mechanisms by examining how the cleverness-RWA relationship operates within its nomological network. For instance, given established links between lower openness and RWA (Butler 2000), a trait consistently associated with creative potential, researchers might test whether openness mediates or moderates the association.

A further important consideration, raised by our use of Bayesian estimation, concerns the role of theory. A key finding is that Bayesian SEM provides more stable parameter estimates in small samples. However, this stability comes with a heightened responsibility for the researcher: the method strongly reinforces the initial model specification. Unlike in large-sample frequentist SEM, where poor fit can prompt exploratory model adjustment, small-sample Bayesian analysis offers limited power to detect model misspecification. Its strength lies not in discovering structure, but in providing precise estimation and uncertainty quantification for a structure that is already well-justified. This underscores that the value of a Bayesian analysis is directly proportional to the strength of the underlying theory.

## 5. Conclusions

This study introduces small-sample-size structural equation modeling to creativity research by applying a step-by-step sample reduction to a model displaying the relationship between creative fluency and cleverness and RWA. Methodologically, we demonstrated Bayesian SEM’s advantages for small-sample modeling in creativity research while establishing practical guidelines: traditional methods suffice for *N* ≥ 150, Bayesian approaches excel at 50–150 cases, and other design choices are needed below N = 50. Our findings highlight that understanding complex psychological constructs, such as DT, requires equally sophisticated analytical approaches, particularly with limited samples, as often found in creativity research.

## Figures and Tables

**Table 1:** Weakly informative priors of the parameters of the Bifactor-(S-1)-model.

Name	Parameter	Prior
nu	item intercept	normal(0,10)
alpha	factor intercept	normal(0,10)
lambda	factor loadings	normal(0,2)
beta	regression coefficients	normal(0,5)
rho	covariances	beta(2,2)
theta	item residual variances	gamma(0.5,0.5)
psi	factor residual variances	gamma(0.5,0.5)

**Table 2:** MLR Estimation Results (N = 198): Standardized Factor Loadings (λ) with Standard Errors, *p*-Values, and 95% Confidence Intervals for Manifest Items of Creative Fluency and Cleverness.

Factor	Indicator	λ	*SE*	*p*	CI Lower	CI Upper
creative fluency	flu_r	0.698	0.054	<.001	0.593	0.803
	flu_p	0.868	0.038	<.001	0.794	0.942
	flu_g	0.842	0.043	<.001	0.759	0.926
	clev_r	0.149	0.081	.065	−0.009	0.307
	clev_p	0.337	0.082	<.001	0.176	0.497
	clev_g	0.198	0.087	.022	0.028	0.369
creative cleverness	clev_r	0.616	0.069	<.001	0.481	0.751
	clev_p	0.679	0.071	<.001	0.540	0.818
	clev_g	0.678	0.070	<.001	0.541	0.815

**Table 3:** Bayesian Estimation Results (*N* = 198): Posterior Distributions for Factor Loadings (Median, Mean) with Convergence Diagnostics (Rhat), Standard Errors, and 95% Credible Intervals.

Factor	Indicator	Rhat	Median	Mean	SE	CI Lower	CI Upper
creative fluency	flu_r	0.999	0.706	0.699	0.045	0.611	0.788
	flu_p	1.000	0.880	0.865	0.035	0.797	0.934
	flu_g	1.001	0.859	0.843	0.036	0.774	0.913
	clev_r	1.000	0.149	0.150	0.076	0.002	0.298
	clev_p	1.001	0.339	0.336	0.071	0.196	0.476
	clev_g	1.000	0.199	0.198	0.074	0.053	0.344
creative cleverness	clev_r	1.000	0.616	0.614	0.062	0.493	0.734
	clev_p	1.000	0.686	0.677	0.062	0.555	0.799
	clev_g	1.000	0.681	0.676	0.063	0.553	0.799

**Table 4:** Fit Indices and Loading Patterns for Frequentist Approach.

Reduction Step	χ^2^(25)	*p*	CFI	RMSEA	SRMR	Range λ Fluency Items	Range λ Cleverness Items	Range λ Nested Cleverness
*N* = 173	29.82	<.01	0.95	0.11	0.04	0.692–0.871	0.142–0.331	0.610–0.675
*N* = 148	24.34	<.01	0.95	0.10	0.04	0.685–0.866	0.135–0.325	0.605–0.670
*N* = 123	25.21	.03	0.94	0.11	0.04	0.678–0.859	0.128–0.318	0.598–0.663
*N* = 98	19.25	.10	0.96	0.09	0.04	0.670–0.850	0.120–0.310	0.590–0.655
*N* = 73 *	17.78	.21	0.95	0.09	0.05	0.655–0.835	0.105–0.295	0.575–0.640
*N* = 48 *	18.15	.16	0.93	0.12	0.06	0.652–0.952	−0.025–0.488	0.530–0.665

Note. * convergence issues were addressed by parameter bounds to constrain variance estimates.

**Table 5:** Standard Error (SE) Inflation Across Progressive Sample Reductions: Comparison of Maximum Likelihood Robust (MLR) and Bayesian (BayesT) Estimation for Fluency and Cleverness.

				*N* = 173				*N* = 148						*N* = 123						*N* = 98						*N* = 73						*N* = 48					
				MLR		BayesT		MLR			BayesT			MLR			BayesT			MLR			BayesT			MLR			BayesT			MLR			BayesT		
Factor		Manifest Item		SE	${∆}_{1}$	SE	${∆}_{1}$	SE	${∆}_{1}$	${∆}_{c}$	SE	${∆}_{1}$	${∆}_{c}$	SE	${∆}_{1}$	${∆}_{c}$	SE	${∆}_{1}$	${∆}_{c}$	SE	${∆}_{1}$	${∆}_{c}$	SE	${∆}_{1}$	${∆}_{c}$	SE	${∆}_{1}$	${∆}_{c}$	SE	${∆}_{1}$	${∆}_{c}$	SE	${∆}_{1}$	${∆}_{c}$	SE	${∆}_{1}$	${∆}_{c}$
creative fluency		flu_r		.059	.005	.048	.003	.061	.002	.007	.050	.002	.005	.070	.009	.016	.056	.006	.011	.075	.005	.021	.062	.006	.017	.118	.043	.064	.075	.013	.030	.118	.000	.064	.084	.009	.039
		flu_p		.041	.003	.036	.001	.041	.000	.003	.036	.000	.001	.048	.007	.010	.039	.003	.004	.054	.004	.014	.045	.006	.010	.097	.043	.057	.051	.006	.016	.093	.000	.051	.061	.010	.026
		flu_g		.046	.003	.037	.001	.048	.002	.005	.039	.002	.003	.052	.004	.009	.042	.003	.006	.060	.008	.017	.045	.003	.009	.100	.040	.057	.053	.008	.017	.100	.000	.057	.069	.016	.033
		clev_r		.085	.004	.081	.005	.091	.006	.010	.090	.009	.014	.106	.015	.025	.099	.009	.023	.115	.009	.034	.112	.003	.026	.136	.021	.055	.131	.030	.056	.149	.013	.068	.167	.036	.092
		clev_p		.086	.004	.076	.004	.094	.008	.012	.084	.008	.012	.106	.008	.020	.093	.009	.021	.119	.013	.033	.103	.010	.031	.161	.042	.075	.122	.019	.050	.147	.014	.089	.157	.035	.085
		clev_g		.091	.004	.081	.005	.101	.010	.014	.088	.007	.012	.111	.010	.024	.080	.000	.006	.125	.014	.028	.110	.030	.048	.147	.022	.050	.129	.019	.067	.171	.024	.074	.167	.038	.105
creative cleverness		clev_r		.074	.005	.069	.007	.080	.006	.011	.075	.006	.011	.088	.008	.019	.080	.005	.016	.098	.010	.029	.092	.012	.028	.112	.014	.043	.107	.015	.043	.145	.033	.076	.124	.017	.060
		clev_p		.076	.005	.068	.006	.081	.005	.010	.073	.005	.011	.090	.009	.019	.078	.005	.016	.101	.011	.030	.087	.009	.025	.123	.022	.052	.106	.000	.017	.146	.023	.075	.118	.012	.029
		clev_g		.075	.005	.068	.005	.082	.007	.012	.074	.006	.011	.080	.002	.014	.080	.006	.017	.102	.022	.036	.091	.011	.028	.114	.003	.039	.111	.000	.027	.142	.028	.067	.119	.008	.035

Note. Items showing incremental (∆_1_) and cumulative (∆_c_) changes in SE. The light grey and white color display indices for one reduction step. For readability leading zeros are not displayed in the table.

**Figure A1:**
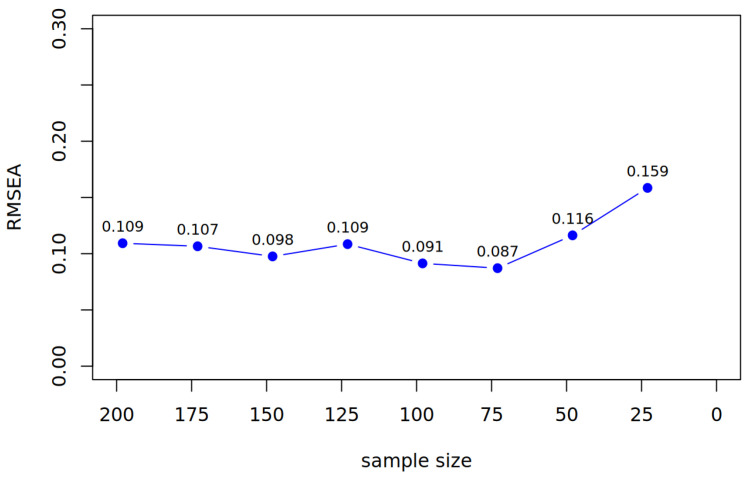
MLR: The change in the RMSEA.

**Figure A2:**
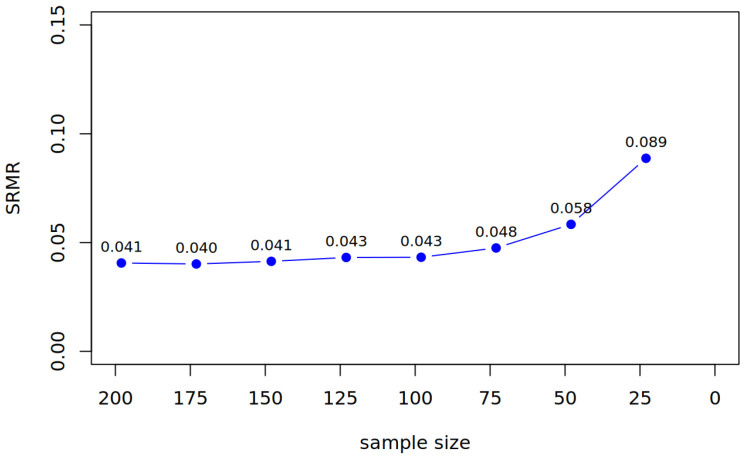
MLR: The change in the SRMR.

**Figure A3:**
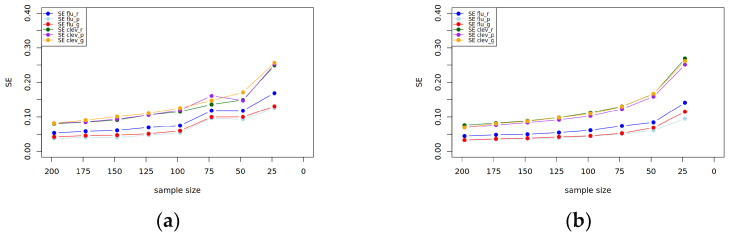
(**a**) MLR: The change in the standard error of the manifest items of Fluency. (**b**) BayesT: The change in the standard error of the manifest items of Fluency.

**Figure A4:**
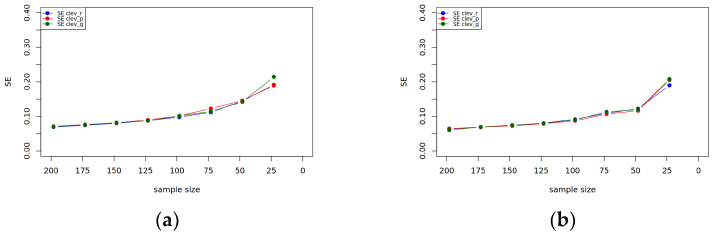
(**a**) MLR: The change in the standard error of the manifest items of Cleverness. (**b**) BayesT: The change in the standard error of the manifest items of Cleverness.

**Figure A5:**
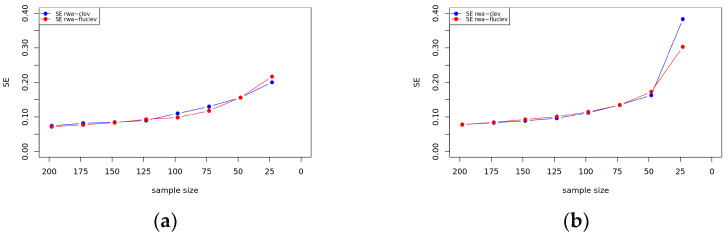
(**a**) MLR: The change in the standard error of the regression coefficients. (**b**) BayesT: The change in the standard error of the regression coefficients.

## Data Availability

The original data presented in the study are openly available in the Open Science Framework (OSF) repository at [https://osf.io/q5ap8]. The original raw data were collected as part of previous studies by (Forthmann et al. 2017, 2020b; Forthmann and Doebler 2022) and can be accessed through their respective publications or by contacting the original authors.

## Abbreviations

The following abbreviations are used in this manuscript:

APA	American Psychological Association
AUT	Alternate Uses Task
CFA	Confirmatory Factor Analysis
CFI	Comparative Fit Index
CI	Confidence/Credibility Interval
DT	Divergent Thinking
MLR	Maximum Likelihood with Robust standard errors
RMSEA	Root Mean Square Error of Approximation
RWA	Right-Wing Authoritarianism
SEM	Structural Equation Modeling
SRMR	Standardized Root Mean Square Residual
MCMC	Markov Chain Monte Carlo
NUTS	No-U-Turn Sampler
HMC	Hamiltonian Monte Carlo
ML	Maximum Likelihood
FIML	Full Information Maximum Likelihood
OSF	Open Science Framework
